# Hypoxia mimetics restore bone biomineralisation in hyperglycaemic environments

**DOI:** 10.1038/s41598-022-18067-1

**Published:** 2022-08-17

**Authors:** Azadeh Rezaei, Yutong Li, Mark Turmaine, Sergio Bertazzo, Christopher A. Howard, Timothy R. Arnett, Kaveh Shakib, Gavin Jell

**Affiliations:** 1 Division of Surgery & Interventional Science, University College London, 9th Floor Royal Free Hospital, London, NW3 2QG UK; 2 Department of Cell & Developmental Biology, University College London, London, WC1E 6BT UK; 3 Department of Medical Physics & Biomedical Engineering, University College London, London, WC1E 6BT UK; 4 Department of Physics & Astronomy, University College London, London, WC1E 6BT UK

**Keywords:** Biomaterials - cells, Biomineralization

## Abstract

Diabetic patients have an increased risk of fracture and an increased occurrence of impaired fracture healing. Diabetic and hyperglycaemic conditions have been shown to impair the cellular response to hypoxia, via an inhibited hypoxia inducible factor (HIF)-1α pathway. We investigated, using an in vitro hyperglycaemia bone tissue engineering model (and a multidisciplinary bone characterisation approach), the differing effects of glucose levels, hypoxia and chemicals known to stabilise HIF-1α (CoCl_2_ and DMOG) on bone formation. Hypoxia (1% O_2_) inhibited bone nodule formation and resulted in discrete biomineralisation as opposed to the mineralised extracellular collagen fibres found in normoxia (20% O_2_). Unlike hypoxia, the use of hypoxia mimetics did not prevent nodule formation in normal glucose level. Hyperglycaemic conditions (25 mM and 50 mM glucose) inhibited biomineralisation. Interestingly, both hypoxia mimetics (CoCl_2_ and DMOG) partly restored hyperglycaemia inhibited bone nodule formation. These results highlight the difference in osteoblast responses between hypoxia mimetics and actual hypoxia and suggests a role of HIF-1α stabilisation in bone biomineralisation that extends that of promoting neovascularisation, or other system effects associated with hypoxia and bone regeneration in vivo. This study demonstrates that targeting the HIF pathway may represent a promising strategy for bone regeneration in diabetic patients.

## Introduction

Diabetes mellitus, a disease presenting with abnormally high levels of blood glucose (hyperglycaemia), can lead to bone metabolism disorders, increasing the risk of fractures, delayed union, the occurrence of non-union fractures and osteoporosis^1^. Clinical studies have reported that fracture healing in diabetic patients is prolonged by 87%^2^. Growing evidence indicates that the role of hyperglycaemia in bone remodelling may be associated with the hypoxia inducible factor (HIF) pathway^3,4^. This oxygen sensing pathway is important for endochondral bone development^5^, bone fracture repair^6^ and bone homeostasis^7^, in addition to a number of steps in fracture repair e.g. inflammatory cell^8^ and bone marrow derived mesenchymal stem cells (BMSCs) recruitment^9^, soft callous formation^5^, bone remodelling^10–12^ and angiogenesis^13^. The role of the HIF pathway in bone biomineralisation is less clear with previous papers demonstrated decreased biomineralisation in hypoxic conditions^7^ but others reporting increased osteoblast proliferation^14^.

In contrast, hyperglycaemia has been shown to decrease the stabilisation of HIF-1α and reduce the expression of hypoxia responsive element (HRE) genes associated with bone regeneration^4,15^. A number of mechanisms for this hyperglycaemic impaired HIF pathway have been suggested (as reviewed by Xiao et al.^16^) including; the inhibition of HIF-1α stabilisation through hyperglycaemia-induced reactive oxygen species (ROS) generation and through advanced glycation end products (AGEs). ROS may increase Ras-related C3 botulinum toxin substrate (Rac1) expression, induce HIF-1α degradation by activating the prolyl hydroxylase domain (PHD) and increase ubiquitin–proteasome activity^17,18^. It has also been reported that hyperglycaemia reduces HIF-1α binding to the HRE and destabilises HIF-1α due to significant homology between the glucose responsive elements and HRE^19^.

Hypoxia plays an important role in bone fracture repair, where microvascular damage following bone fracture causes a hypoxic environment^20^ and HIF-1α stabilisation^21^. This causes HIF-1α mediated inflammatory/angiogenic factor production and initiation of the inflammatory phase of fracture repair vital for normal bone regeneration^8,22^. Hypoxia and HIF-1α stabilisation has been shown to be important in BMSC recruitment^23^, BMSC proliferation^24^, regulation of BMSC differentiation into chondrocytes and osteoblasts^22^. HIF-1α has been shown to upregulate a plethora of pro-angiogenic genes by cells associated with fracture repair (e.g. VEGF and bFGF)^25,26^. During the hard callus remodelling phase where ordered bone is formed, HIF stabilisation has also been shown to be important for osteoclastogenesis, osteoclast function and coupling osteoblast- osteoclast cross talk^21^.

Artificial stabilisation of HIF-1α may offer a therapeutic approach for improving bone regeneration in diabetic patients and has gained attention in the field of bone tissue engineering. Indeed, HIF stabilising chemicals (chemicals that inhibit the degradation of HIF-1α at normal oxygen level) such as cobalt chloride (CoCl_2_), dimethyloxalylglycine (DMOG) and desferrioxamine (DFO) have been reported to stabilise HIF-1α in numerous cell types, including osteoblasts^27–29^ and promote fracture repair in vivo in normal glucose levels^30,31^. The effect of HIF stabilisation on bone regeneration and biomineralisation in hyperglycaemic conditions is, however, less clear. CoCl_2_, DMOG and DFO target HIF pathway via distinct mechanisms. CoCl_2_ appears to stabilise HIF-1α by competing with iron ions (Fe^2+^), binding to the PHD-2 active site ^32^. DFO downregulates PHD-2 and factor inhibiting HIF (FIH) activity via Fe chelation, due to their dependence on this ion^33^,whereas DMOG competes with 2-oxoglutarate and binds to both PHD-2 and FIH^34^.

In vitro studies have demonstrated an effect of hyperglycaemia on osteoblasts extending that of HIF mediated inhibition of angiogenesis. Balint et al*.*^35^ and Pereira et al*.*^36^ studies reported glucose-inhibition of in vitro bone biomineralisation. Another study by Terada et al*.*^37^ on osteoblast-like cells (MG-63) revealed that hyperglycaemia impaired the polyol-sorbitol pathway and intercellular sorbitol accumulation appeared to be associated with inhibited biomineralisation.

Whilst the HIF pathway is undoubtably important in angiogenic signalling and restoring the vasculature in bone repair, the direct role of HIF-1α on osteoblast behaviour and biomineralisation in both normal and hyperglycaemic conditions is uncertain. Furthermore, there may be differing effects between the of lack of oxygen (hypoxia) and HIF-1α stabilisation on bone regeneration. Using an in vitro bone nodule formation model from neonatal rat calvarial osteoblasts^38^ and a multidisciplinary characterisation approach including biological, ultrastructural and microstructural quantitative techniques, this study investigates the role of hypoxia (1.0% O_2_) and two HIF stabilisers CoCl_2_ and DMOG at normoxia (20% O_2_), in normal (5.5 mM) and increasing glucose concentrations (25 mM and 50 mM) glucose conditions. This will enable us to determine whether targeting HIF pathway can promote bone formation in hyperglycaemic environments.

## Results

### Hyperglycaesmia inhibited nodule formation

**Figure 1:**
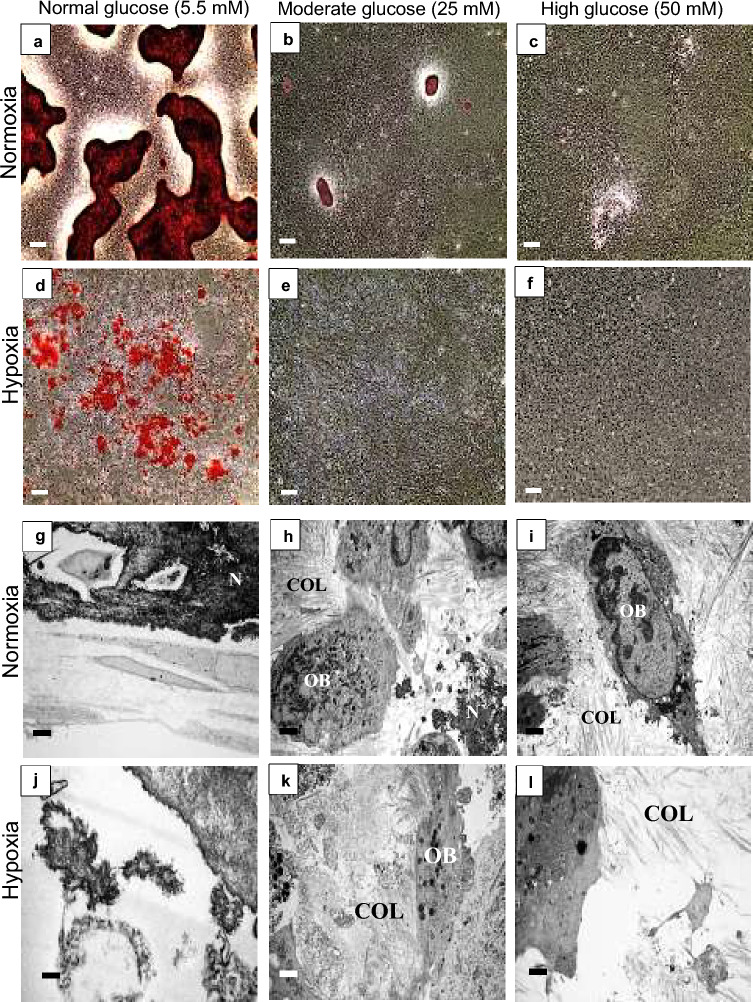
Hypoxia (1% O_2_) and hyperglycaemia inhibited bone nodule formation. After 21 days, Alizarin Red stained (ARS) dense nodules were observed in (**a**) normal (5.5 mM) glucose, whilst (**b**) moderate (25 mM) and (**c**) high (50 mM) glucose conditions inhibited nodule formation. (**d**) Hypoxia normal glucose showed discrete biomineralisation that was not associated with collagen fibres. (**e**) Moderate and (**f**) high glucose inhibited biomineralisation. Transmission electron microscopy (TEM) micrographs of (**g**) normoxia normal glucose, (**h**) moderate glucose and (**i**) high glucose showed a glucose concentration dependent inhibition of bone nodule formation. COL fibres were not observed in (**j**) hypoxia normal glucose, but (**k**) moderate and high glucose environments in hypoxia showed some collagen fibres. Scale bar for (**a**–**f**) is 200 µm and for (**g**–**i**) is 2 µm. (n = 5) (N: nodule, COL: collagen fibres, OB: osteoblast).

Mature nodules, with well-defined edges were observed in normoxia (20% O_2_) normal glucose concentration (5.5 mM) (Fig. 1a), with mineralised extra-cellular collagen fibres as determined with TEM (Fig. 1g). However, moderate (25 mM) and high glucose (50 mM) environments inhibited nodule formation in a concentration dependant manner. Only a few smaller bone nodules were detectable in moderate glucose levels (Fig. 1b), with very few areas of extra-cellular mineral evident, whilst high glucose completely inhibited nodule formation (Fig. 1c). Collagen fibres and few mineralised structures were observed in TEM micrograph of moderate glucose (Fig. 1h), while high glucose only exhibited collagen fibres and nodules were hardly detectable in this condition (Fig. 1i).

Hypoxia (1% O_2_) in normal glucose concentrations, completely prevented organic matrix deposition and the formation of bone structures (Fig. 1d). Biomineralisation deposition in hypoxic cultures was limited to small discrete calcified sites that were not associated with extracellular matrix (ECM-collagen fibres), this is sometimes referred to as dystrophic biomineralisation (Fig. 1d,j). SEM micrographs also confirmed presence of nodules that are higher than the culture surface in normoxia in normal glucose and dystrophic biomineralisation in hypoxia (Supplementary Fig. 1a,b). Neither nodules nor biomineralisation were observed under hypoxia moderate (Fig. 1e) and high (Fig. 1f) glucose conditions, although TEM revealed abundant collagen fibres in these conditions (unlike hypoxia low glucose where no collagen fibres were present) (Fig. 1k,l).

**Figure 2:**
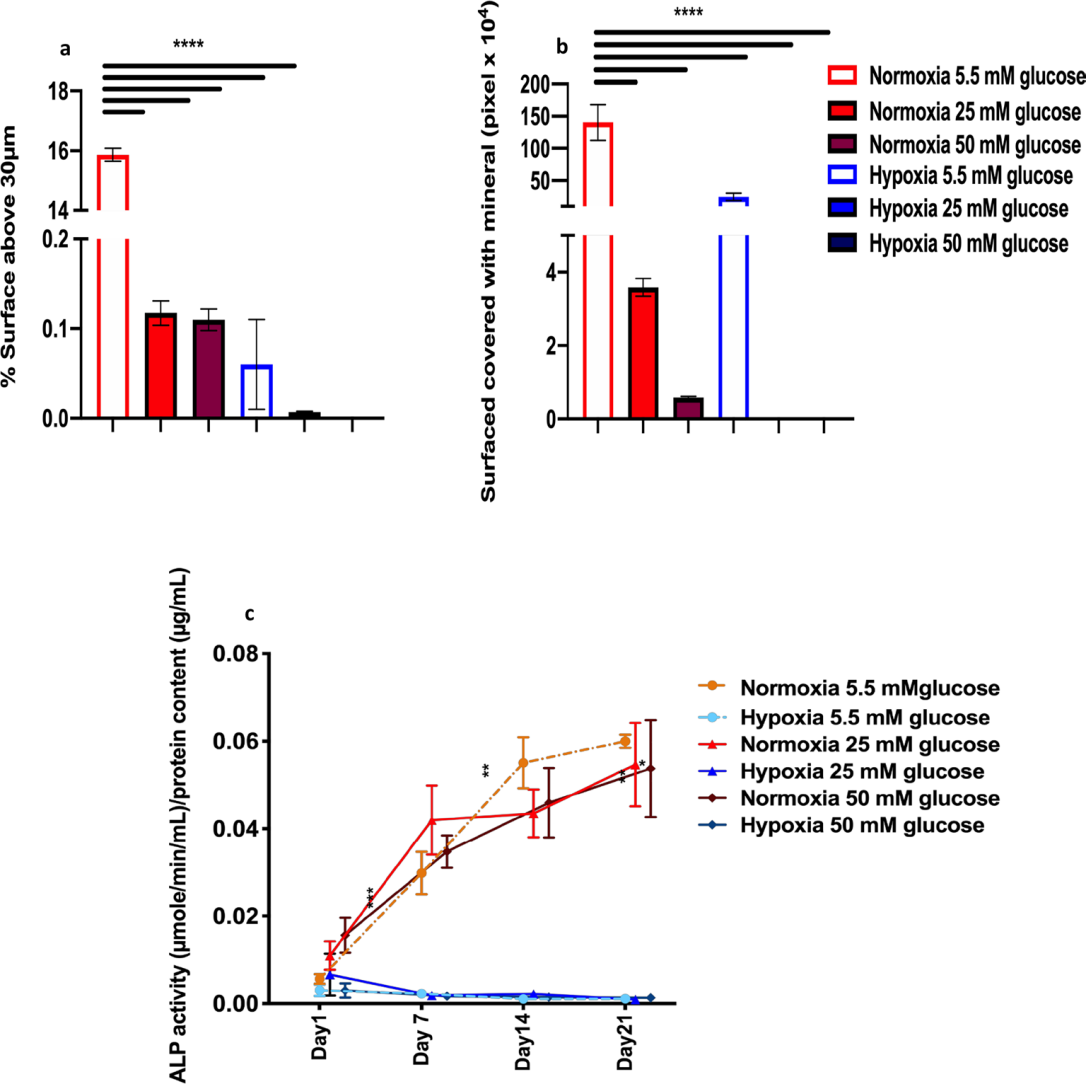
Inhibition of bone nodule formation in hypoxia and hyperglycaemia quantified by (**a**) interferometry (area above 30 µm) and (**b**) image analysis of Alizarin Red staining (total area). Nodules cultured in normoxia normal (5.5 mM) glucose covered a substantially larger area (**a** and **b** total area) than both normoxia moderate (25 mM) and high (50 mM) glucose. (**c**) ALP activity per unit protein revealed that all normoxic conditions had higher ALP production than hypoxic conditions. Error bars represent the SD from the mean values. *P ≤ 0.05; **P ≤ 0.01; ***P ≤ 0.001 (n = 4).

Quantification of bone nodule size was determined using interferometry (Fig. 2a) and 2D image quantification of mineralised area (Fig. 2b) which confirmed the morphological observations with Alizarin Red stain (ARS). Nodules formed in normoxia normal glucose were larger (with 15.9 ± 0.2% of the surface above 30 µm, Fig. 2a) and covered more area than the discrete biomineralisation observed in hypoxia normal glucose (only 0.1 ± 0.1% of the surface above 30 µm, Fig. 2b, Supplementary Fig. 1c,d). Moderate and high glucose conditions showed a decrease in nodule formation compared to normoxia normal glucose (Fig. 2a). A larger mineralised area was observed in hypoxic conditions measured using image quantification, compared to interferometry (Fig. 2a,b), this is expected considering the numerous small discrete mineralised dystrophic nodules as opposed to the raised, mineralised ECM (bone nodules) observed in normoxia and highlights the caution needed in interpreting area quantification of biomineralisation alone without volume or nodule size measurements (e.g. with interferometer analysis) or ultrastructure characterisation.

Hypoxia radically reduced ALP expression (P ≤ 0.001) in all glucose environments (Fig. 2c). In normoxia high glucose condition, increased ALP expression (P ≤ 0.001) after day 1 but a decreased expression in ALP was observed in both moderate and high glucose conditions after 14 days in culture (P ≤ 0.01and P ≤ 0.05). All glucose levels showed similar ALP activity after 21 days.

### CoCl_2_ and DMOG restored bone nodule formation in hyperglycaemic environments

To investigate the effects of HIF-1α stabilisation on bone formation in hyperglycaemic conditions, osteoblasts were treated with the hypoxia mimetic CoCl_2_ and DMOG for 21 days. The effect of CoCl_2_, DFO and DMOG concentrations on osteoblasts metabolic activity and proliferation were initially performed. DFO significantly inhibited cell proliferation at all concentrations (12.5–50 µm) and was therefore excluded from future experiments.

**Figure 3:**
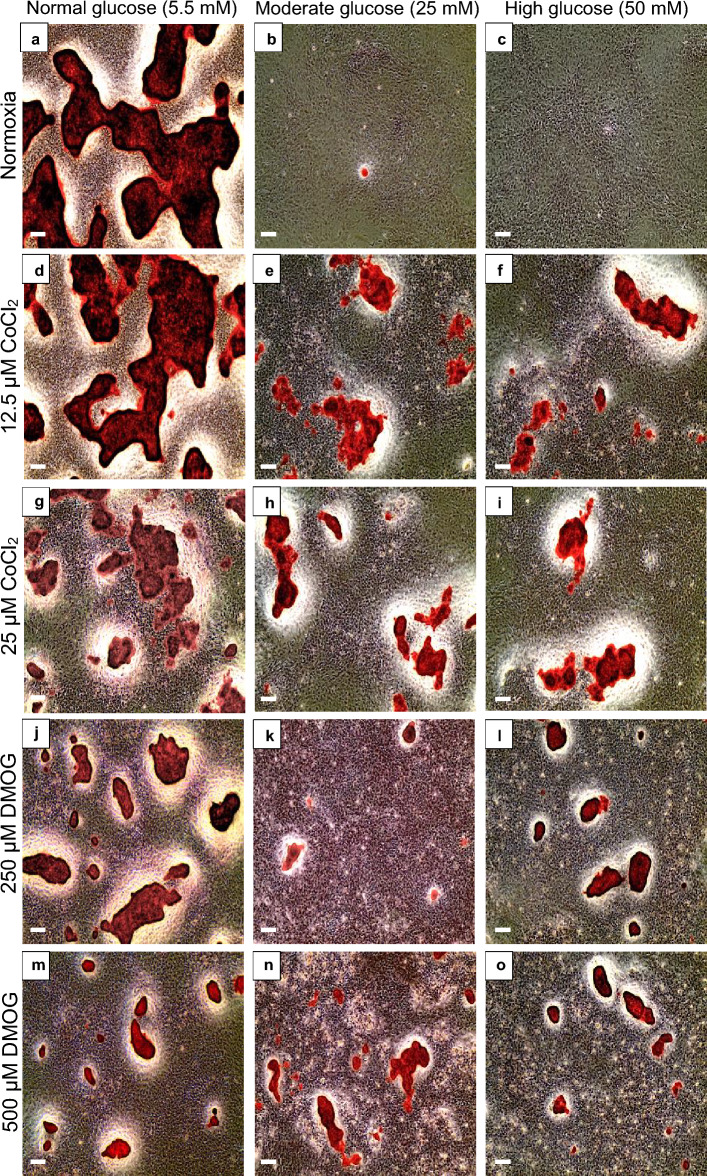
CoCl_2_ and DMOG restored nodule formation in hyperglycaemic cultured osteoblasts. After 21 days, Alizarin Red staining (ARS) showed bone nodule formation in untreated cultures in (**a**) normal glucose (5.5 mM), whilst the addition of (**b**) moderate (25 mM) and (**c)** high glucose (50 mM) inhibited nodule formation. (**d**–**o**) Cultures treated with hypoxia mimetics CoCl_2_ and DMOG decreased bone nodule formation in normal glucose levels but increased bone nodule formation in moderate and high glucose levels compared to untreated controls. Scale bar for all images is 200 µm. (n = 5).

**Figure 4:**
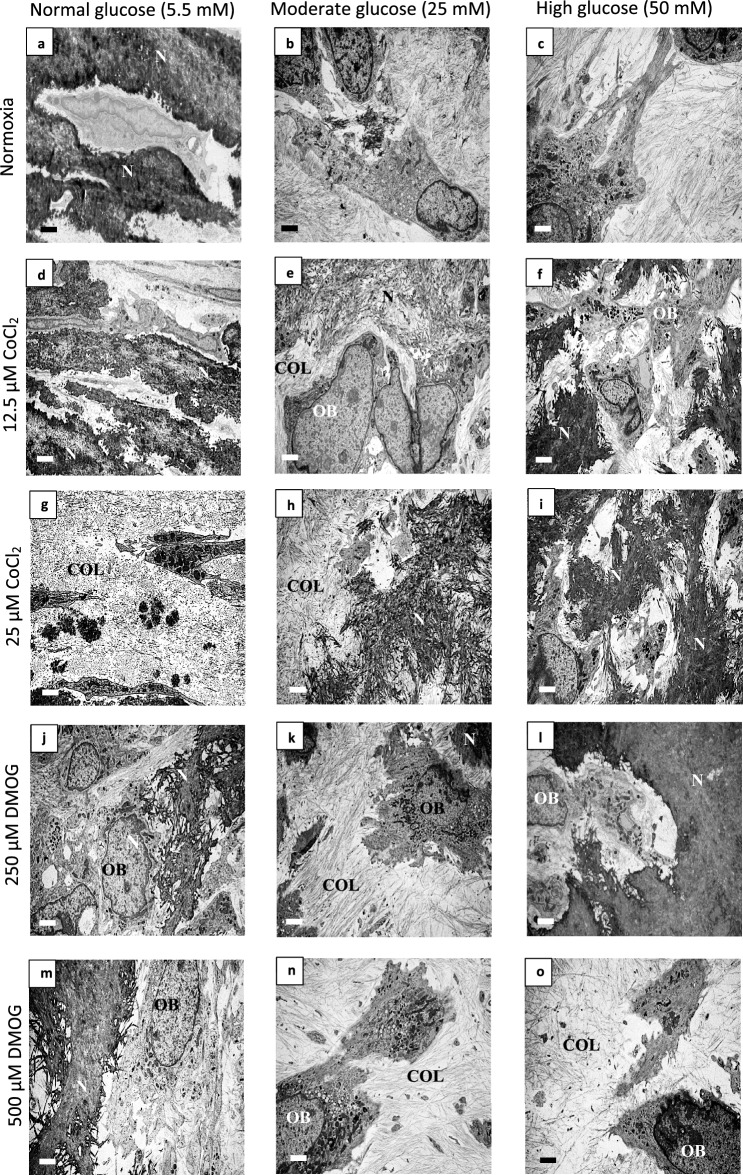
The effects of HIF-1α mimetics and differing glucose environments on the ultrastructure of bone nodules. Transmission electron microscopy (TEM) of (**a**–**c**) moderate and high glucose levels showed reduced extracellular mineralised collagen fibres compared to normal glucose in normoxia. The HIF-1α mimetics (**d**,**j**) CoCl_2_ and (**j**,**m**) DMOG, reduced extracellular mineralised collagen in normal (5 mM glucose conditions) but restored extracellular (bone-like) mineral in (**e**,**k**,**h**) moderate (25 mM) and (**f**,**i**,**l**) high (50 mM) glucose conditions compared to (**a**–**c**) untreated controls (with the exception of **o**) 500 mM DMOG where no extracellular mineralised collagen fibres were observed. Scale bar for all images is 200 µm. (n = 5) (N: nodule, COL: collagen fibres, OB: osteoblast).

**Figure 5:**
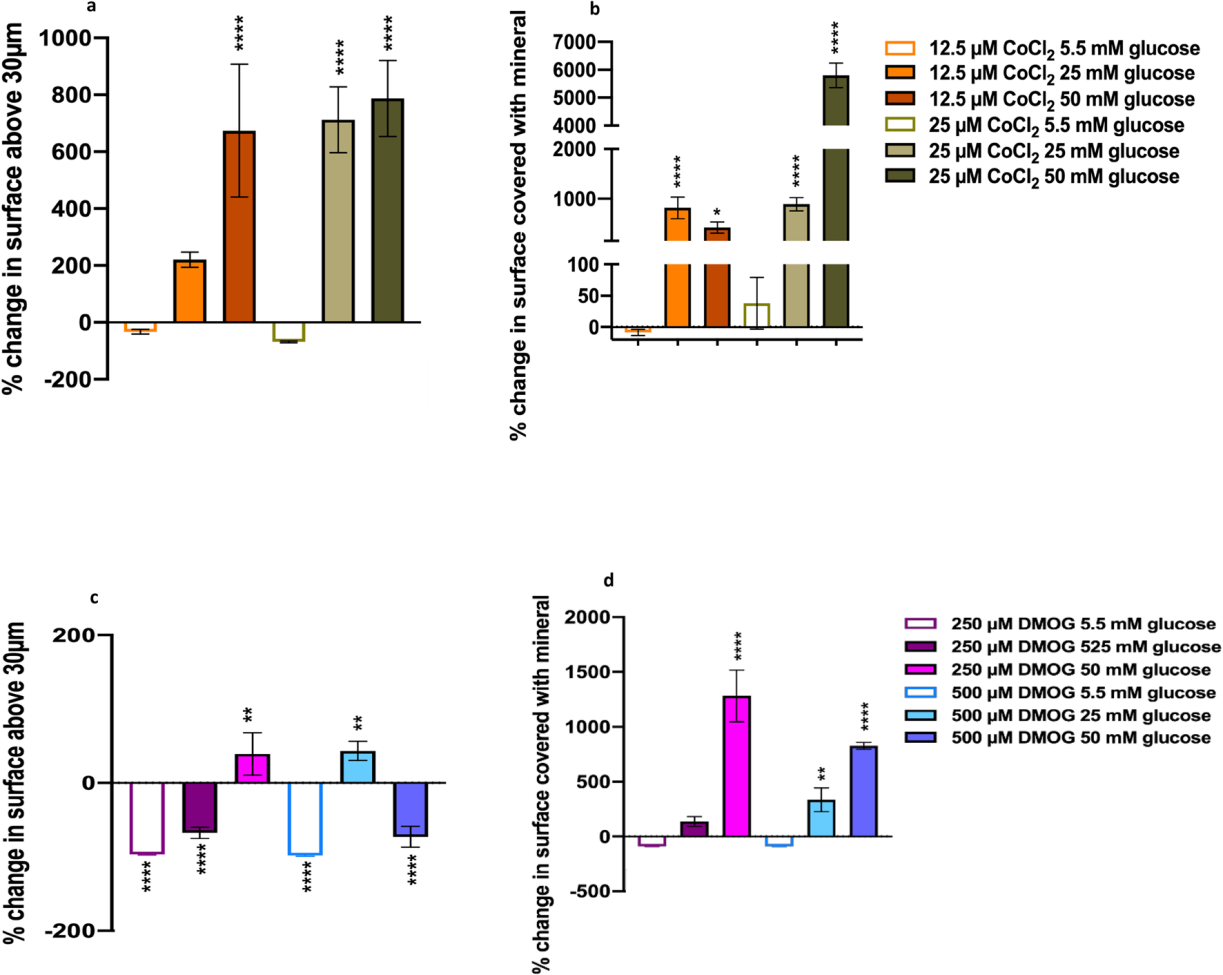
Quantification of bone nodules restored by CoCl_2_ and DMOG treatment in hyperglycaemic conditions using interferometry and ImageJ. (**a**) The percentage change (percentage change compared corresponding glucose levels in atmospheric O_2_) in the surface covered with nodules (above 30 µm compared to untreated control (similar level of glucose) without CoCl_2_ as quantified with interferometry and (**b**) 2D image analysis of area of surface covered by mineral. Both 12.5 µM CoCl_2_ and 25 µM CoCl_2_ recovered nodule formation in moderate (25 mM) and high (50 mM) environments. (**c**) DMOG interferometry and (**d**) Image J analysis also revealed that both 250 µM DMOG and 500 µM DMOG restored nodule formation, however, DMOG exhibited a reduced restoration capacity compared to CoCl_2_. Error bars represent the SD from the mean values. *P ≤ 0.05; **P ≤ 0.01; ****P ≤ 0.0001 (n = 4).

Both CoCl_2_ and DMOG restored bone nodule formation in hyperglycaemic environment as observed by ARS (Fig. 3), mineralised ECM (Fig. 4) and quantification (Fig. 5). Addition of both 12.5 µM CoCl_2_ and 25 µm CoCl_2_ to normoxia normal glucose (Fig. 3d,g), did not prevent bone formation (as observed in hypoxia normal glucose (Fig. 1d)) and distinct nodules were present as observed by ARS (Fig. 3d,g). Lighter ARS regions in cells cultured in 25 µM CoCl_2_ normal glucose (Fig. 3g) indicated less dense calcium content compared with that of 12.5 µM CoCl_2_ normal glucose (Fig. 3d) and normoxia normal glucose (Fig. 3a), where darker red structures were visible. CoCl_2_ concentrations above 25 µM (normal glucose), completely inhibited bone formation in all conditions (supplementary Fig. 2a–c). Notably treatment of the cells with the HIF-1α stabilising factor 12.5 µM CoCl_2_ and 25 µM CoCl_2_ restored nodule formation in moderate (Fig. 3e,h) and high hyperglycaemic conditions (Fig. 3f,i) compared to untreated conditions (Fig. 3b,c). Although the size of the nodules was smaller than those in normoxia normal glucose (Fig. 3a).

In a similar manner to CoCl_2_, the HIF-1α stabilising factor DMOG (250 µm and 500 µM), also promoted nodule formation in both moderate (Fig. 3k,n) and high (Fig. 3l,o) hyperglycaemic culture conditions compared to untreated hyperglycaemic controls (Fig. 3b,c). DMOG (250 µM and 500 µM) did not inhibit nodule formation in normal glucose level (Fig. 3j,m) but formed smaller nodules compared to CoCl_2_ treated (Fig. 3d,g) and untreated conditions (Fig. 3a). DMOG concentrations above 500 µM had an inhibitory effect on bone nodule formation at all glucose levels (Supplementary Fig. 2d–f).

### The effect of hypoxia mimetics and hyperglycaemia on bone ultrastructure

Increased glucose levels completely inhibited ECM biomineralisation in normoxia as observed by TEM ultrastructure (Fig. 4a–c). CoCl_2_ and DMOG did not prevent extracellular biomineralisation (Fig. 4). Differences were observed, however, between CoCl_2_ and DMOG at differing concentrations. The mineralised ECM of the CoCl_2_ and DMOG treated cultures in normal glucose (Fig. 4d,g,j,m) were less electron dense (not so dark), compared to the untreated culture (Fig. 4a), possibly indicating less mature biomineralisation. In moderate glucose conditions, 25 µM CoCl_2_ appeared to be less densely mineralised (Fig. 4g) compared to both 12.5 µM CoCl_2_ and untreated normal glucose cultured (Fig. 4a). Interestingly, although extracellular biomineralisation was observed in both 12.5 µM CoCl_2_ and 25 µM CoCl_2_ in moderate and high glucose conditions, the nodules restored in moderate glucose did not seem to be as mature compared to those in high glucose conditions (Fig. 4e,h compared to f and i). Abundant collagen fibres were observed in all hypoxia mimetic treated samples, but a reduction of number and organisation was observed in 500 µM DMOG treated cells in high glucose.

### Quantification of bone nodule formation

In hyperglycaemia conditions, the HIF-1α mimetics (CoCl_2_ and DMOG) increased bone nodule size (percentage of surface area with nodules above 30 µm) and area compared to untreated controls with similar level of glucose (Fig. 5). Whilst in normal (5.5 mM) glucose conditions, CoCl_2_ had no significant effect on nodule formation, DMOG decreased the size of the nodules (area above 30 µM, P ≤ 0.001).

In hyperglycaemic conditions, the level of bone nodule HIF-1α mediated increase depended upon the type and concentration of HIF-1α mimetic, and the glucose environment. CoCl_2_ had a bigger influence on nodule formation, in terms of the size of the nodules (Fig. 5a,c) and area (Fig. 5b,d) than DMOG. CoCl_2_ increased the area of nodules above 30 µm in all hyperglycaemic conditions by (200–790%), DMOG only showed an increase of 39% and 43% in 250 µm in HG and 500 µm DMOG in MG, respectively (Fig. 5c). While treatment with CoCl_2_ significantly increased bone nodule formation in both moderate and high glucose conditions, it is important to note that it did not restore nodule formation to the same level as normal glucose controls.

### The effect of hypoxia mimetics (DMOG and CoCl_2_) on ALP expression

The HIF stabilising factor, CoCl_2_ decreased ALP activity in all glucose conditions on day 1 (compared to untreated conditions) but did not affect the expression of ALP at later time points (day14, 21). On day 7, 12.5 µm CoCl_2_ normal glucose and 25 µM CoCl_2_ high glucose exhibited significantly enhanced ALP activity compared to the untreated controls (Supplementary Fig. 3a). The percentage change in ALP activity also showed that DMOG decreased ALP activity in all glucose conditions on day 1 (compared to untreated conditions) but did not affect the expression of ALP at later time point (day21). On day 7, 250 µM DMOG high glucose and on day 14, both 250 µm DMOG and 500 µM DMOG in moderate and high glucose exhibited significantly increased ALP activity compared to the untreated controls (Supplementary Fig. 3b).

### Compositional analysis of the bone nodules

**Figure 6:**
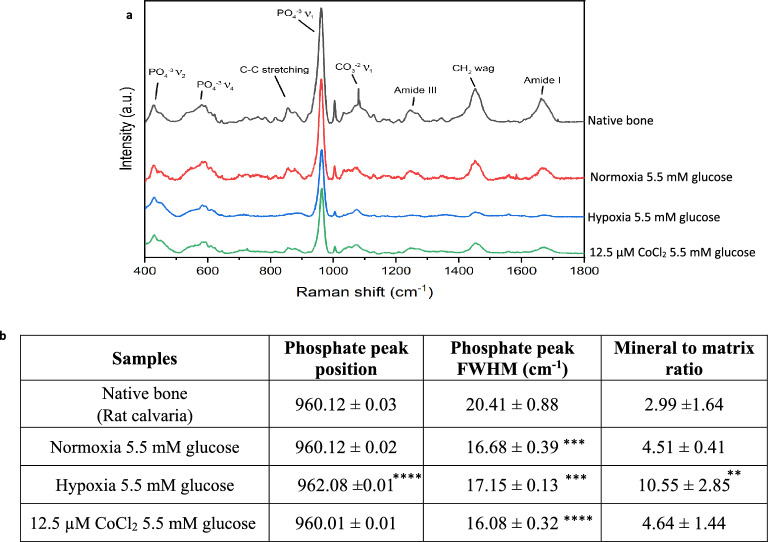
Raman spectra of rat calvarial native bone and rat calvarial osteoblasts in normoxia, hypoxia and 12.5 µM CoCl_2_ all in normal glucose conditions (5.5 mM). (**a**) Average Raman peaks associated with proteins (Amide III, CH_2_, Amide I) are much reduced in hypoxia compared to normoxia, (**b**) with a much higher mineral to matrix ratio. The hypoxia mimetic CoCl_2_ had a different effect than hypoxia and had a biochemical composition more similar to normal culture conditions and bone. **P ≤ 0.01; ***P ≤ 0.001*; ****P ≤ 0.0001 (the minimum number of bone nodules per treatment = 10).

Raman spectroscopy was also performed to assess the biomolecular composition of the bone nodules (Fig. 6a,b). The nodules formed in normoxia normal glucose and those treated with CoCl_2_ had a similar mineral to matrix ratio (PO_4_^−3^ ν_1_ area : amide III) to the native bone, and similar position of the phosphate peak (~ 960 cm^−1^), while hypoxia normal glucose exhibited a more crystalline non-substituted hydroxyapatite as indicated by phosphate peak position (~ 962 cm^−1^) (P ≤ 0.0001) and a much increased mineral: matrix ratio (P ≤ 0.01). This marries with the ultrastructure (TEM) and morphological observations of dystrophic biomineralisation in hypoxic conditions, where there is much reduced protein contribution to the small crystalline minerals formed (i.e., in hypoxia there is not mineralised extracellular collagen fibres as observed in normoxia and CoCl_2_ conditions). Unfortunately, DMOG and hyperglycaemic conditions Raman spectral analysis wasn’t possible due to the smaller number and smaller size of the nodules available.

### Hyperglycaemia impairs the HIF pathway and downregulates VEGF expression

**Figure 7:**
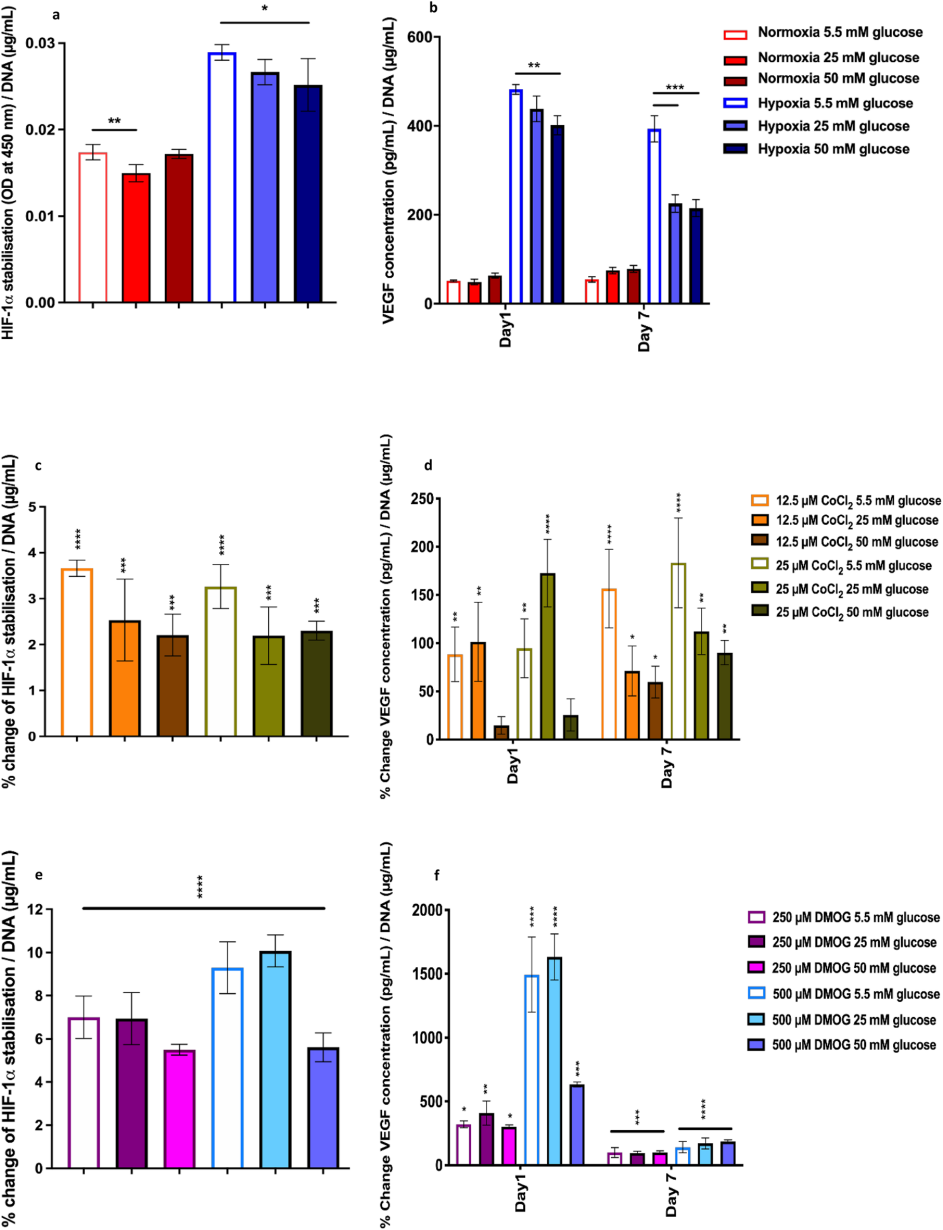
HIF-1α stabilisation on day 3 and VEGF response on day 1 and 7 of osteoblast to hypoxia and hypoxic mimetics CoCl_2_ and DMOG in hyperglycaemic conditions. (**a**) Hyperglycaemia impaired HIF pathway and (**b**) decreased VEGF expression. (**c**–**f**) Both CoCl_2_ and DMOG restored HIF-1α stabilisation and VEGF expression in hyperglycaemic conditions (percentage change compared corresponding glucose levels in atmospheric O_2_). Error bars represent the SD from the mean values. *P ≤ 0.05; **P ≤ 0.01; ***P ≤ 0.001*; ****P ≤ 0.0001 (n = 4).

Hypoxia increased HIF-1α stabilisation (Fig. 7a) and VEGF expression (Fig. 7b) in normal glucose conditions. Hyperglycaemia decreased HIF-1α stabilisation (Fig. 7a) and VEGF expression (Fig. 7b) in response to hypoxia after 1 day (P ≤ 0.05). Interestingly, moderate but not high glucose conditions also decreased HIF-1α stabilisation in normoxia (Fig. 7a). Prolonged culture in hyperglycaemic conditions further decreased the osteoblastic response to hypoxia with reduced VEGF expression by 42% and 45% compared to normal glucose hypoxic response (Fig. 7b).

### CoCl_2_ and DMOG stabilise HIF-1α and increase VEGF

CoCl_2_ and DMOG increased HIF-1α stabilisation in all glucose conditions (Fig. 7c,d respectively). The amount of HIF-1α stabilised by the hypoxia mimetics were less than observed in hypoxia (1% O_2_) but caused substantial increases in VEGF expression. High glucose conditions decreased the hypoxia mimetic stabilisation capability (P ≤ 0.001 in CoCl_2_, P ≤ 0.0001 in DMOG) and the VEGF response for both DMOG and CoCl_2_ on day 1 compared to untreated controls. Both HIF-1α mimetics increased VEGF expression in all glucose environments compared to their normoxic controls (Fig. 7e,f), with the exception of VEGF expression in response to CoCl_2_ in high glucose environments on day 1. DMOG increased VEGF expression substantially more than CoCl_2_ and to a similar level observed in response to hypoxia on day 1 for 250 µM DMOG, and considerably exceeding the hypoxia response with 500 µM DMOG treatment. On day 7, whilst still significant, there was reduced VEGF expression in response to hypoxia mimetics in cultures exposed to DMOG compared to day 1.

## Discussion

Patients with diabetes have decreased bone regeneration capacity and cells cultured in hyperglycaemic conditions (25 and 50 mM) have been demonstrated to have an impaired HIF-1α pathway and a consequent reduced ability to respond to hypoxic conditions^39^. Here we have demonstrated that bone nodule formation is inhibited by both hypoxia (1% O_2_) and hyperglycaemia (in a glucose concentration dependent manner). Furthermore, we have demonstrated, for the first time, that HIF stabilising molecules (CoCl_2_ and DMOG) can restore bone nodule formation in hyperglycaemic conditions.

The majority of in vitro studies use a range of glucose between 25 and 50 mM^36,37,40,41^, which is much higher than in diabetic patients, where the term hyperglycaemia is used for blood glucose concentrations above 70–110 mg/dL (3.9–6.1 mM)^42^. The justification for the use of these high levels in vitro, is to replicate cell behavioural responses (including reduced regenerative responses to hypoxia^13,14^) caused by the long-term consequences of impaired glucose metabolism in vivo.

In this study we used atmospheric O_2_ pressure as “normoxia”, this is clearly far from the normal oxygen levels found in bone, as reviewed by Stuart et al.^43^. The oxygen level in a healthy human femur has been reported to be ~ 9.4% and upon fracture, it reduces to 1.54%^44,45^. In a fracture haematoma of a rabbit model, 0.8% O_2_ and 3.8% O_2_ were observed 2 days and 4 weeks post fracture, respectively^46–48^. Whilst mimicking the in vivo environment is undoubtedly important for in vitro modelling, it must also be considered that in vitro there isn’t a multicellular and systemic response to hypoxia, as observed in vivo. Indeed an in vitro study by Utting et al*.* found that atmospheric oxygen pressures of 20% formed the most bone compared to lower oxygen pressures (with inhibition of nodule formation in 12% and 5%, strong inhibition at 2% and 1% and complete inhibition in 0.2%)^49^. A key component of tissue engineered models is to demonstrate the ability to form 3D tissue that is similar to the native tissue. This model demonstrated the ability to form mineralised ECM (TEM Fig. 1), that is biochemically similar to native bone (Raman Spectroscopy Fig. 6) and in sufficient volumes that differences in glucose levels were quantifiable with interferometer measurements (Fig. 5).

### Hypoxia inhibits bone formation but causes aberrant extracellular biomineralisation

In agreement with previous studies, our results demonstrated that hypoxia (1% O_2_) in normal culture conditions (5.5 mM glucose) reduced mineralised bone nodule formation^10,49^. Utting et al.^49^ reported slight inhibition of nodule formation in 12% and 5% O_2_, strong inhibition in 2% and 1% O_2_ and complete inhibition in 0.2% O_2_. Our results demonstrate, through ultrastructural (TEM), biochemical (Raman spectroscopy) and size quantification (interferometry measurement), that these hypoxic nodules (or mineralised structures) were morphologically, and biochemically different to the control bone nodules or native bone. Despite covering a large percentage of the surface, the discrete mineralised structures were smaller (less than 30 µm in height), more crystalline, and had a much higher mineral:matrix ratio (P ≤ 0.01) than that observed in native bone or the control nodules (Fig. 6a). Ultrastructural analysis (TEM) found few collagen fibrils present in the hypoxic cultures (compared to the abundant fibres present in other oxygen tensions) and importantly the mineral structures were not associated with collagen fibrils. A decrease in collagen in hypoxic conditions has been mentioned in previous studies^11,50^ and this lack of collagen may be because of the oxygen dependent functionality of the procollagen prolyl 4-hydroxylase, an enzyme involved in collagen formation^51^. The mineral formed in hypoxic structures is therefore not bone, but discrete hydroxyapatite mineral. It is unclear if these discrete mineral structures are formed spontaneously (possibly due to the addition of calcium and phosphate supplements commonly added to bone cultures) or is osteoblast mediated.

In addition to the reduced collagen (discussed above) other causes for hypoxia mediated impaired bone formation have been previously discussed including; the reduced proliferation and differentiation of immature osteoblasts^49^, and the reduction of soluble proteins (e.g. ALP, osteocalcin) important in osteoblast differentiation and biomineralisation^50,52,53^. In accordance with this literature, our results showed that hypoxia dramatically decreased osteoblast ALP activity and cell number in all glucose conditions^54^.

The hypoxic biomineralisation, observed here, illustrates the caution that must be used when interpreting the ARS as an indicator of in vitro bone formation. As illustrated by our results, ARS identifies calcium rich deposits, rather than a mineralised extracellular matrix found in bone. This may be especially relevant if high concentration of calcium and phosphate are present in the bone culture (e.g., through the addition of calcium and phosphate supplements, or the release of these ions by bioceramics). In contrast to bone biomineralisation, calcification is not a bone specific characteristic and can occur in other body tissues such as arteries^55^.

### The difference between HIF-1α stabilisation and hypoxia on bone nodule formation

Hypoxia (when cultured in normal 5.5 mM glucose) caused a substantial and prolonged decrease in ALP activity, together with a reduction in collagen and small dystrophic or discrete mineralised structures. However, treatment with HIF-1α stabilising factors (CoCl_2_ and DMOG), revealed a different response, where extracellular mineralised collagen fibres were observed and ALP activity was only temporarily decreased (on day 1). This suggests that oxygen availability may affect HIF-1α-independent cellular mechanisms important in extracellular biomineralisation. There are a number of cellular responses to low oxygen pressure that are independent of the HIF pathway and occur prior to HIF-1α transcriptional response, these include glycolysis regulation (prior to upregulation of glycolytic enzymes and glucose transporters via HIF-1α), ROS production/regulation and the pre-transcriptional inhibition of oxygen sensitive proteins (such as the PHDs involved collagen synthesis).

The differences in biomineralisation observed between hypoxia and hypoxia mimetic stabilisation could also reflect the differing specificity and sensitivity of hypoxia mimetics (cobalt and DMOG), in targeting the HIF-1α pathway compared to hypoxia. For example, it has been reported that cobalt can upregulate HIF-1α genes whilst also down-regulating HIF-2α genes^56^, whilst hypoxia upregulates both. This may be particularly important in bone biomineralisation where HIF-2α stabilisation has recently been shown to inhibit osteoblast differentiation^57^. Likewise differing oxygen pressure environments, and different durations of hypoxic exposure, have been shown to have different effects on osteoblastogenesis and bone formation^50^. Deletion of the HIF-1α pathway would help elucidate the differing effect of the chemical hypoxia mimetics and hypoxia, but may be technically challenging for in vitro bone models (requiring long-term culture), where inhibition of the pathway decreases osteoblast differentiation (osteocalcin expression)^58^ and increases oxidative stress^28^. This may be why (to date) bone formation has not been demonstrated in vitro with HIF-1α inhibited primary osteoblasts.

Our results demonstrate that the use hypoxia mimetics can have favourable outcomes, compared to hypoxia, for bone tissue engineering, where high numbers of cells are needed (hypoxia mimetics did not inhibit proliferation compared to hypoxia), and bone-like nodule formation is possible.

### Hyperglycaemia reduces bone nodule formation

Hyperglycaemic conditions (25 and 50 mM) impaired the HIF-1α pathway (as observed with the reduced HIF-1α stabilisation and decreased expression of VEGF in response to hypoxia) and dramatically reduced bone nodule formation. Hyperglycaemia is known to cause HIF-1α dysfunction^59,60^ and the impairment of bone formation in diabetic patients^36,61^. In vivo, this is likely to be due to the consequential decreased in angiogenesis^52^, osteoblast-osteoclast cross talk dysfunction^53^, reduced MSC recruitment^62^, changed inflammatory response^63^ and other system effects. Here we show that hyperglycaemia inhibits biomineralisation in vitro, and this is in accordance with a few other reports where it is suggested that hyperglycaemia inhibits osteoblast proliferation and differentiation^40,64^.

The intracellular mechanism of how hyperglycaemia inhibits biomineralisation remains uncertain. In vitro, the glucose dependant impairment of the HIF-1α did not appear to effect osteoblast collagen formation, as observed with abundant expression in TEM images. Another study has also reported similar results, where high glucose (25 mM) did not diminish collagen production^65^. Likewise, whilst ALP activity, was drastically reduced in hypoxia it was not clearly affected by hyperglycaemia in our results. Others have reported a glucose concentration dependent effect on ALP production. Cunha et al*.*^66^ and Gopalakrishnan et al.^54^ reported that hyperglycaemia (16.5–49.5 mM) reduced ALP activity, whilst Garcia-Hernandez demonstrated that 12 mM glucose enhanced ALP activity^40^. Differences in cell type, duration of experimentation and ALP normalisation approaches may account for why the glucose concentration ALP inhibitory effects were not observed in our cell culture. The differences observed between hypoxia (non-ECM associated minerals) and high glucose levels (abundant collagen but no biomineralisation) on bone formation, suggests that glucose dependant inhibition of bone formation involves different mechanisms than hypoxia.

### HIF stabilising chemicals CoCl_2_ and DMOG restore bone nodule formation in hyperglycaemic environments

Here, for the first time, we demonstrated that artificial stabilisation of HIF-1α (with CoCl_2_ and DMOG) promoted bone nodule formation in hyperglycaemic conditions. The intracellular mechanism for this restoration is unclear. In vivo, hyperglycaemic impaired bone formation is often explained as due to the impaired angiogenic response in diabetic tissues (as seen here where hyperglycaemia reduced VEGF expression)^67^, or due to the increased ROS production promoting osteoclastogenesis^68^. HIF-1α mimetic restoration or enhanced bone formation are thought to be due to increased angiogenesis (as seen in Fig. 7) and restoration of osteoblast-osteoclast balance^29,69^. This clearly cannot be the explanation of the hypoxia mimetic restoration of bone-like extracellular matrix seen here (Figs. 3 and 4), where a osteoblast cell-line model was used. Rather, it suggests that that hypoxia may affect HIF-1α independent pathways important in bone mineralisation (possibly pre-transcription changes e.g. in ROS availability or glycolysis). Alternatively, it may mean that the hypoxia mimetics are also affecting other HIF independent pathways, due to their likely interaction with other oxygen sensitive Fe containing enzymes (e.g. procollagen prolyl 4-hydroxylase)^51^.

It is interesting to speculate on the mechanism of hypoxia mimetic restoration of bone mineralisation in hyperglycaemia, and a possible explanation could include the reduction of ROS through HIF-1α mediated generation of ROS scavengers, and modification of glycolysis^70^. Interestingly, Munoz-Sanchez et al*.* reported that hyperglycaemia and actual hypoxia impaired mitochondrial function^71^, whilst CoCl_2_ treatment prevented mitochondria damage^72^. Mitochondrial damage may also inhibit mitochondrial related biomineralisation, which has been previously observed in osteoblasts^73,74^. Although this doesn’t fully explain inhibition of extracellular mineralisation. Another explanation of why hypoxia mimetic agent restored hyperglycaemic inhibited bone formation, could be due to hypoxia mimetic agent activation of osteogenic differentiation factors. HIF-1α stabilisation has been shown to increase the gene expression of osteogenic differentiation factors such as Runt-related transcription factor (RUNX)-2 and osteocalcin^75^, although others have reported that hypoxia (as opposed to hypoxia mimetic agents) decreases RUNX-2 and osteocalcin expression^76^. A further explanation, related to metabolic adaptation, is the effects of the hypoxia mimetic agents on osteoblast number and differentiation. Osteoblast number is known to effect in vitro mineralisation^49^. Our analysis of proliferation (DNA quantification supplementary Fig. 4) showed that hypoxia mimetics did not cause significant change in the proliferation compared to normoxia (compared with the same level of glucose), whilst hypoxia did. Thus, differentiation might be the cause for the hypoxia mimetics mediated restoration. It is of course possible, due the broad mode of action of the hypoxia mimetics, that the hypoxia mimetic agents (Co and DMOG) are influencing HIF independent pathways, and that these are also important in restoring bone nodule formation in hyperglycaemia.

### Hypoxia mimetics for treatment of diabetic related bone disorders

HIF stabilisation strategies need to control the location and duration of HIF stabilisation, as systemic HIF stabilisation or uncontrolled HIF stabilisation may cause chronic inflammation and other adverse effects^77–79^. The development of HIF stabilising materials would help control the release. Bioactive glasses (BGs) are a group of silica-based inorganic biomaterials with the ability to form carbonated hydroxyapatite layer with exposure to biological fluids which facilitates binding of BGs to bone surface and bone regeneration^80,81^. Creating BGs with different ion release profiles e.g., silicate (Si), strontium (Sr) or cobalt (Co), for specific patient cohorts (e.g. for diabetic patients) is not only possible but probably favourable in the evolution of this field.

## Conclusion

A hyperglycaemic bone model was developed that demonstrated that moderate and high glucose levels (25 and 50 mM) inhibit bone nodule formation. Interestingly, for the first time, it was demonstrated that the use of hypoxia mimetics (CoCl_2_ and DMOG) helped restore bone nodule formation. This discovery demonstrates the role of the HIF pathway in bone development and the separate roles of HIF-1α stabilisation and low oxygen availability. Targeting the HIF-1α pathway in bone, offers the possibility of new interventions, including the design of biomaterials or tissue scaffolds specifically for patients with impaired bone regeneration due to a defective cellular oxygen sensing pathway.

## Materials and methods

### Bone nodule formation

Calvarial osteoblastic cells were isolated from 3-day-old Sprague–Dawley rats according to the sequential enzyme digestion protocol described by Orriss et al.^38^. All animal experimentation protocols were approved by the University College London (UCL) Animal Care Services Ethical Review Committee, licensed under the UK Home Office regulations and carried out in accordance with the UK Animal (Scientific Procedures) Act 1986 (Home Office, London, United Kingdom) upholding the highest standards of ethical practice and care in all aspects of this research. This study was carried out in compliance with the ARRIVE guidelines (https://arriveguidelines.org).

Cells were seeded at a density of 6 × 10^4^ cells/well into 12-well plates in Alpha modified minimum essential medium (α-MEM) supplemented with 10% foetal bovine serum (FBS; Thermo Fisher Scientific), 100 U/mL penicillin, 100 µg/mL streptomycin, 0.25 µg/mL amphotericin (Sigma-Aldrich) and 2 mM l-glutamine (Life Technologies) and kept at 37 °C in a humidified normoxia incubator under 20% O_2_ and 5% CO_2_ (Binder GmbH, Germany). To facilitate removal of the nodules from the wells for further microscopy quantification, 21 mm diameter Melinex disks (thickness: 175 µM; Agar Scientific) were used as a substrate for cell seeding. When confluent (3 days), cells were exposed to 5.5, 25 and 50 mM glucose-conditioned supplemented α-MEM containing 2 mM β-glycerophosphate, 10 nM dexamethasone, and 50 μg/mL ascorbate (all from Sigma-Aldrich). Cells were then treated with either 12.5, 25 or 50 µM CoCl_2_ (Sigma-Aldrich) or 250, 500 or 1000 µM DMOG (Sigma-Aldrich) with 5.5, 25 and 50 mM glucose and kept at 37 °C in normoxic conditions for 21 days. The medium was exchanged for fresh medium every 2–3 days.

Initially, the effect of CoCl_2_, DMOG and DFO on osteoblasts metabolic activity (AlamarBlue assay, Invitrogen) and proliferation (DNA quantification assay, Sigma) were tested. DFO significantly inhibited cell proliferation at all concentrations (12.5–50 µm) and was therefore excluded from future experiments. Normoxia (20% O_2_) and hypoxia (1% O_2_, 5% CO_2_, Innova CO-48 hypoxia incubator, New Brunswick Scientific, USA) with 5.5, 25 and 50 mM glucose conditioned mediums were used as controls.

### Angiogenic response

On day 1 and 7, the cell culture supernatants were collected and spun using a plate centrifuge (Hettich Universal 320R, Germany) at 1500 rpm for 5 min at 4 °C to remove cellular debris. VEGF concentration was quantified using a Quantikine ELISA kit (R & D Systems, UK) according to the manufacturers’ protocol. VEGF concentration was normalised to DNA content.

### HIF-1α stabilisation

Osteoblasts were seeded into T75 flasks at a density of 1.2 × 10^6^ in 15 ml supplemented α-MEM and treated with the conditions mentioned above. After 72 h, nuclear extracts were prepared using the nuclear extraction kit (Abcam, UK) according to the manufacturer’s instructions. The protein activity of HIF-1α was measured in nuclear extracts using HIF-1α transcription factor assay kit (Abcam, UK). HIF concentration was normalised to DNA content.

### Alkaline phosphatase (ALP) activity

On day, 1, 3, 7, 14 and 21 ALP activity (Alkaline Phosphatase Assay Kit; Abcam) was determined using the manufacturer’s protocol, by mixing 50μL of osteoblasts cell lysates (as explained in previous section) with 50μL of 5 mM pnitrophenyl phosphate (pNPP). After 60 min, when the colour change occurred, the assay was stopped by adding 20μL of the stop solution (NaOH), and the absorbance was measured at 405 nm using a multimode microplate reader (TECAN Infinite M200 PRO, Switzerland). ALP activity was normalised to total protein content determined using BCA protein assay kit (Merck).

### Characterisation of bone nodules

#### Alizarin Red staining (ARS) assay

After 21 days culture, the cells were washed with PBS, fixed in 4% paraformaldehyde (PFA; Sigma-Aldrich) for 15 min, washed again and incubated with 40 mM Alizarin Red stain (ARS-pH between 4.1 and 4.5) in ddH_2_O for 20 min with gentle shaking on a shaker. ARS was removed, cells were washed five times with ddH_2_O and air dried. An EVOS XL Core light microscope (Thermo Fisher Scientific, UK) was used to take images of stained cells at 10× magnification.

#### Structural characterisation using transmission electron microscopy (TEM)

Day 21 nodules were fixed in freshly made 2% PFA and 1.5% glutaraldehyde in a 0.1 sodium cacodylate buffer (both from Sigma-Aldrich) and kept in 4 °C fixative solution in refrigeration overnight. Nodules were then post-fixed in 1% osmium tetroxide and 1.5% potassium ferrocyanide (both from Sigma-Aldrich), suspended in 0.1 M cacodylate buffer and maintained at refrigerated storage for 90 min. The samples were then washed with 0.1 M cacodylate buffer and distilled deionised water respectively. Specimens were dehydrated in a graded ethanol water series at concentrations of 25%, 50%, 70% and 90% for 5 min each and in 100% for 4 × 5 min. The cultures were infiltrated with Agar100 epoxy resin and propylene oxide mix (mixture 1:2, 1:1 and 2:1 Agar Scientific) for an hour each, and with pure epoxy resin for 4 h. The samples were then prepared for sectioning by laying the Melinex disc cell side down on to a resin filled beam capsule and hardened at 60 °C for 48 h. Representative areas were selected and sections of 70–80 nm, were cut using a diamond knife on a Reichert ultra-cut S microtome (Leica, Milton Keynes, UK). The sections were collected on Formvar slot copper grids (300 mesh), stained with lead citrate (Sigma-Aldrich), and viewed with a JEOL 1010 transition electron microscope (TEM; Tokyo, Japan) operated at 100 kV. Images of the samples were then recorded using a Gatan Orius CCD camera.

#### Surface analysis by scanning electron microscopy (SEM)

Day 21 nodules were fixed, post fixed and dehydrated as mentioned in TEM section. The dehydrated samples were then mounted on aluminium stubs with carbon tape, sputter coated with a thin layer of gold measuring approximately 10 nm and viewed in an environmental electron microscope (ESEM; FEI Quanta 200 FEG, Netherlands) using voltage of 15 kV.

#### 3-Dimensional analysis of bone nodules using interferometry

Day 21 nodules, were fixed in 4% PFA at room temperature for 15 min, rinsed three times with ddH_2_O and air-dried at room temperature overnight. For each sample, the nodules at the centre of the Melinex disc (36 mm^2^ square-shaped area; Agar Scientific) were assessed with Nexview-NX2 3D optical interferometer (Zygo, Middlefield, CT, USA) using 2.75× objective lenses, 0.5× zoom and a scan length of 145 µm. A minimum of 4 wells per treatment were analysed. The height and area covered with nodules (above 20 µm) were analysed in Mx software. The minimum was height was chosen to differentiate between spontaneous calcification (as observed in hypoxia) and mineralised ECM which is raised from the cell surface. The ARS images of were also used to measure the 2D surface covered with nodules in Image J (U.S. National Institutes of Health, Bethesda, MD, USA).

#### Biochemical analysis of bone nodules using Raman spectroscopy

Osteoblasts were cultured on magnesium fluoride substrate (MgF_2_) (Crystran, UK) which is a weak Raman scatterer within the biochemical range of interest and does not interfere with the results. On day 21, the MgF_2_ disks were rinsed with ddH_2_O and air dried at room temperature overnight. Air dried native bones from 3-day-old SD rat calvaria were used as controlled specimens for comparison. Spectra were collected using a 785 nm laser, 50 mW laser power, at room temperature and atmospheric pressure, on a Renishaw inVia spectrometer equipped with a Leica microscope. The laser was focused onto the sample using a 20X objective lens, ~ 3–4 µm diameter spot size. The spectra were recorded at a resolution of ~ 1–2 cm^−1^ in the region of 300–2000 nm, with 30 s acquisition time per spectrum and repeated 3 times per spot. A minimum of 10 bone nodules were sampled from 3 different MgF_2_ for each treatment. The Raman spectra were processed and analysed with the Origin 9pro (OriginLab Corporation, Northampton, MA, USA), according to the protocol suggested by Gentleman et al.^82^. To assess the biochemical properties of the bone nodules univariate spectral analyses was performed to measure the average area of three peaks: apatite phosphate (PO_4_^–3^ ν_1_) symmetric stretch (near ~ 960 cm^−1^), the type B υ_1_ CO_3_ peak (~ 1070 cm^−1^), the amide I band (~ 1665 cm^−1^). The mineral to matrix ratio was also determined by dividing the PO_4_^–3^ ν_1_ band area by the matrix band area (amide I). The full width half maximum of the phosphate (PO_4_^–3^ ν_1_) symmetric stretch (near ~ 960 cm^−1^), as a measure of mineral crystallinity, was also determined. and compared to native bone.

### Statistical analysis

All analyses were calculated using GraphPad Prism 8 software (CA, USA) unless otherwise stated. A minimum of 3 experimental repeats were considered for analyses. Significant differences between conditions were determined using the Student’s-t-test with Welch’s corrections for comparison between two data sets and one-way ANOVA followed by Holm-Sidak’s multiple comparisons test for more than two data sets, with significance being defined as a p-value of less than 5% (P < 0.05).

### Ethical approval

All animal experimentation protocols were approved by the University College London (UCL) Animal Care Services.

## Supplementary Information

Supplementary Figures.

## Data Availability

The datasets used and/or analyzed during the current study are available from the corresponding author on reasonable request.
