# Author Correction: Hypoxia mimetics restore bone biomineralisation in hyperglycaemic environments

**DOI:** 10.1038/s41598-025-17199-4

**Published:** 2025-09-10

**Authors:** Azadeh Rezaei, Yutong Li, Mark Turmaine, Sergio Bertazzo, Christopher A. Howard, Timothy R. Arnett, Kaveh Shakib, Gavin Jell

**Affiliations:** 1 Division of Surgery & Interventional Science, University College London, 9th Floor Royal Free Hospital, London, NW3 2QG UK; 2 Department of Cell & Developmental Biology, University College London, London, WC1E6BT UK; 3 Department of Medical Physics & Biomedical Engineering, University College London, London, WC1E6BT UK; 4 Department of Physics & Astronomy, University College London, London, WC1E 6BT UK

Correction to: *Scientific Reports* 10.1038/s41598-022-18067-1, published online 17 August 2022

The original version of this Article contained errors in Figures 1 and 3.

As a result of an error during figure assembly, incorrect images were displayed for the condition Hypoxia – high glucose (50 mM) in Figure 1f and for 25 µM CoCl_2_ – high glucose (50 mM) in Figure 3.

The original Figures 1 and 3 and accompanying legends appear below.

**Fig. 1:**
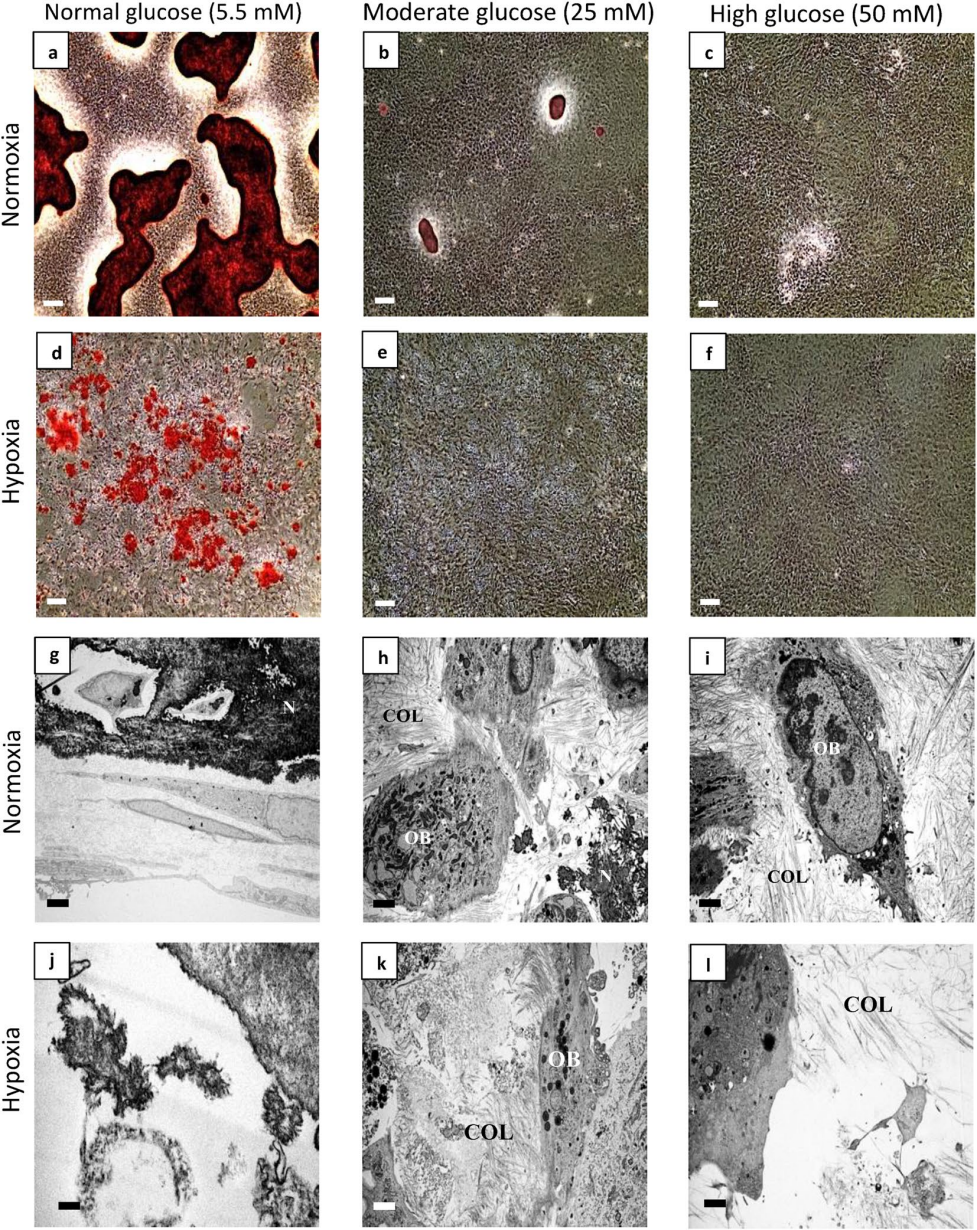
Hypoxia (1% O_2_) and hyperglycaemia inhibited bone nodule formation. After 21 days, Alizarin Red stained (ARS) dense nodules were observed in (**a**) normal (5.5 mM) glucose, whilst (**b**) moderate (25 mM) and (**c**) high (50 mM) glucose conditions inhibited nodule formation. (**d**) Hypoxia normal glucose showed discrete biomineralisation that was not associated with collagen fibres. (**e**) Moderate and (**f**) high glucose inhibited biomineralisation. Transmission electron microscopy (TEM) micrographs of (**g**) normoxia normal glucose, (**h**) moderate glucose and (**i**) high glucose showed a glucose concentration dependent inhibition of bone nodule formation. COL fibres were not observed in (**j**) hypoxia normal glucose, but (**k**) moderate and high glucose environments in hypoxia showed some collagen fibres. Scale bar for (**a–f**) is 200 µm and for (**g–i**) is 2 µm (n = 5) (N: nodule, COL: collagen fibres, OB: osteoblast).

**Fig. 3:**
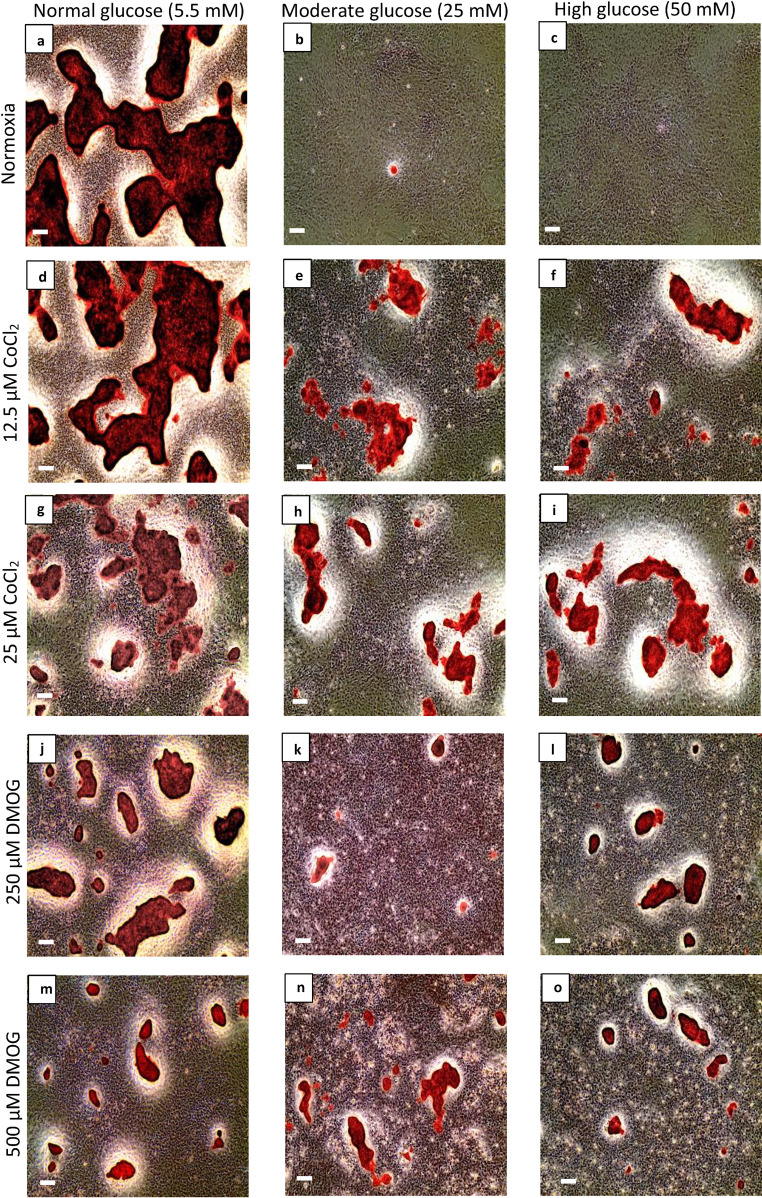
CoCl_2_ and DMOG restored nodule formation in hyperglycaemic cultured osteoblasts. After 21 days, Alizarin Red staining (ARS) showed bone nodule formation in untreated cultures in (**a**) normal glucose (5.5 mM), whilst the addition of (**b**) moderate (25 mM) and (**c**) high glucose (50 mM) inhibited nodule formation. (**d–o**) Cultures treated with hypoxia mimetics CoCl_2_ and DMOG decreased bone nodule formation in normal glucose levels but increased bone nodule formation in moderate and high glucose levels compared to untreated controls. Scale bar for all images is 200 µm (n = 5).

The original Article has been corrected.

