# Supplementary material for: Hypoxia mimetics restore bone biomineralisation in hyperglycaemic environments
Source: Sci Rep. 2022 Aug 17;12:13944. doi: 10.1038/s41598-022-18067-1 (PMC9385857; doi:10.1038/s41598-022-18067-1)
Supplement: Supplementary file 1 — Supplementary Figures. [file 41598_2022_18067_MOESM1_ESM.docx]

**Title: Hypoxia mimetics restore bone biomineralisation in hyperglycaemic environments**

**Azadeh Rezaei^1^, Yutong Li^1^, Mark Turmaine^2^, Sergio Bertazzo^3^, Christopher A Howard^4^, Timothy R Arnett^2^, Kaveh Shakib^1*^, Gavin Jell^1*^**

Azadeh Rezaei: [a.rezaei@ucl.ac.uk](mailto:a.rezaei@ucl.ac.uk) , Yutong Li: [yutong.a.li@ucl.ac.uk](mailto:yutong.a.li@ucl.ac.uk) , Mark Turmaine: [m.turmaine@ucl.ac.uk](mailto:m.turmaine@ucl.ac.uk) , Sergio Bertazzo: [s.bertazzo@ucl.ac.uk](mailto:s.bertazzo@ucl.ac.uk) , Christopher A Howard: [c.howard@ucl.ac.uk](mailto:c.howard@ucl.ac.uk) , Timothy R Arnett: [t.arnett@ucl.ac.uk](mailto:t.arnett@ucl.ac.uk) , Kaveh Shakib: [k.shakib@ucl.ac.uk](mailto:k.shakib@ucl.ac.uk) , Gavin Jell: [g.jell@ucl.ac.uk](mailto:g.jell@ucl.ac.uk)

^1^Division of Surgery & Interventional Science, University College London, 9^th^ Floor Royal Free Hospital, NW3 2QG, UK

^2^Department of Cell & Developmental Biology, University College London, WC1E 6BT, UK

^3^Department of Medical Physics & Biomedical Engineering, University College London, WC1E 6BT, UK

^4^ Department of Physics & Astronomy, University College London, WC1E 6BT, UK

***Corresponding authors:**

Prof Gavin Jell Prof Kaveh Shakib

Div of Surgery & Interventional Science Div of Surgery & Interventional Science

9th Floor Royal Free Hospital 9th Floor Royal Free Hospital

London, NW3 2QG, UK London, NW3 2QG, UK

Email: [g.jell@ucl.ac.uk](mailto:g.jell@ucl.ac.uk) Email: [k.shakib@ucl.ac.uk](mailto:k.shakib@ucl.ac.uk)

Tel: 02080168016 Tel: 02080168016

**Supplementary Figures**

Hypoxia

Normal glucose (5.5 mM)

Normoxia

Normal glucose (5.5 mM)

**
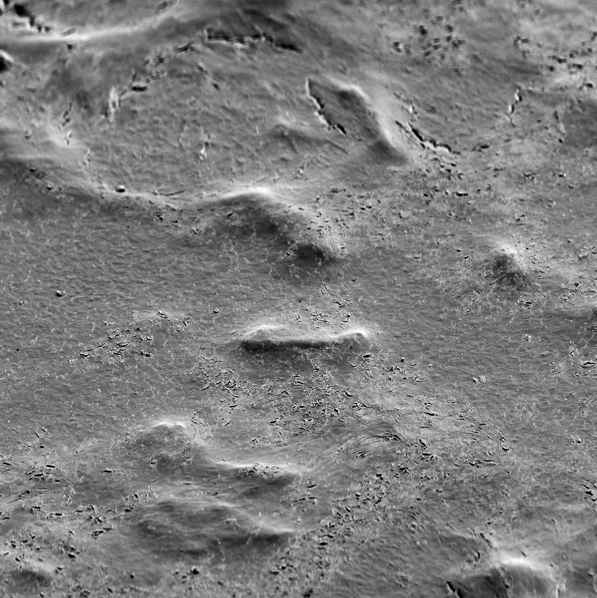

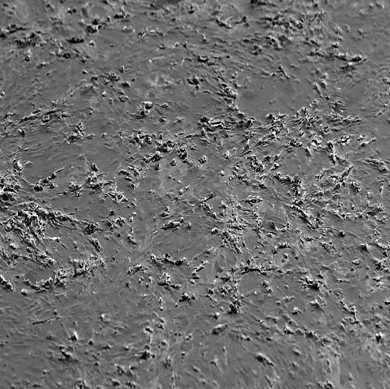
**

**a**

**b**

**
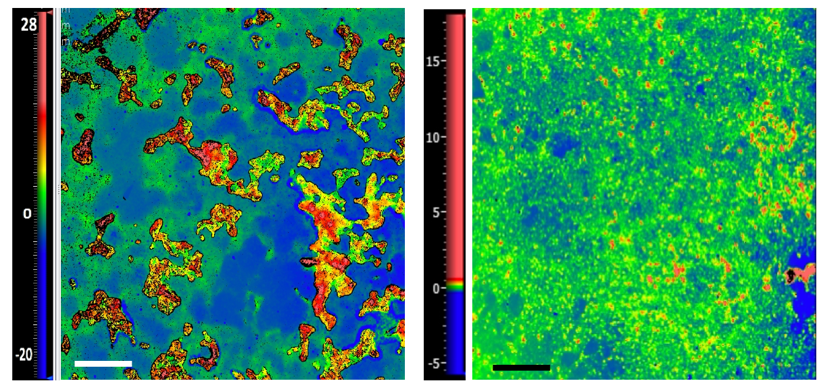
**

**d**

**c**

**Figure 1.** Progressive inhibition of nodule formation under hypoxic (1% O_2_) condition. On day 21, scanning electron microscopy (SEM) images showed large nodules raised from the surface in **a** normoxia (20% O_2_) normal glucose (5.5 mM) and dystrophic mineralisation in hypoxia normal glucose. Phase map of **c** normoxia and **d** hypoxia normal glucose confirmed bone formation inhibition under hypoxic condition. (Scale bar for a & b is 200 μm and for c & d is 1mm)

High glucose (50 mM)

Moderate glucose (25 mM)

Normal glucose (5.5 mM)


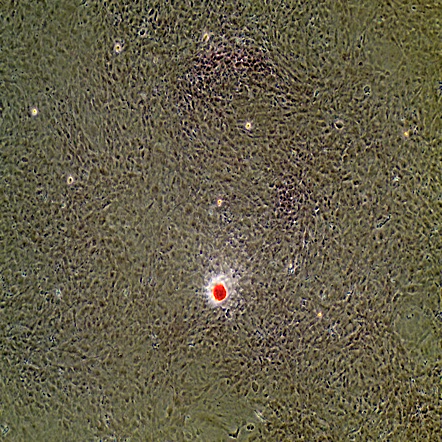

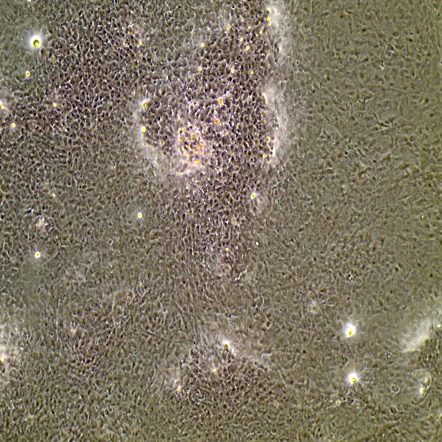
**
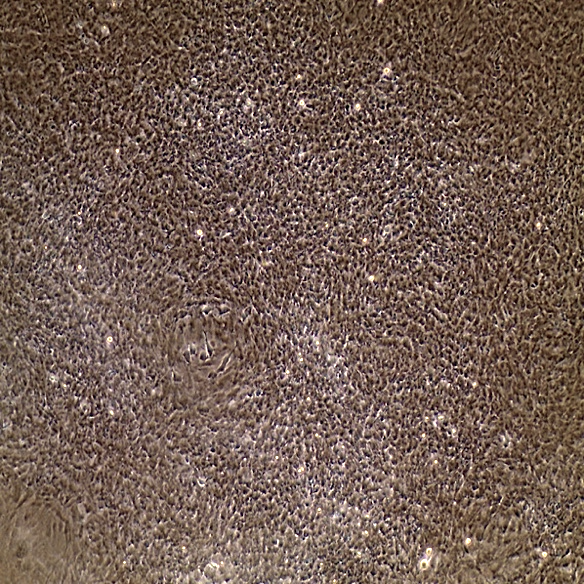
**

**b**

**c**

**a**

50 µM CoCl_2_

**
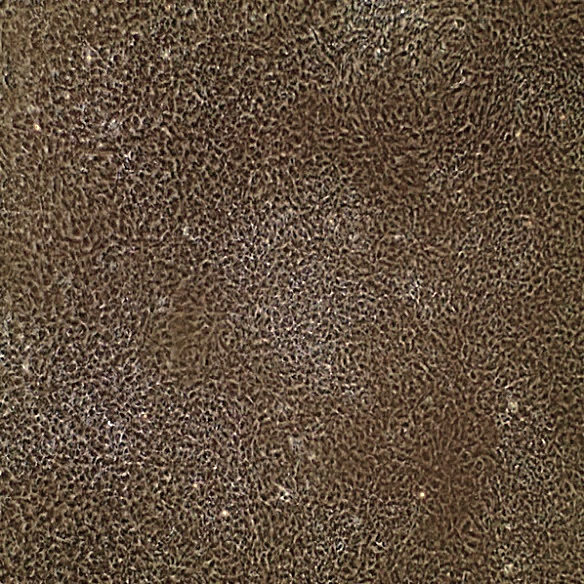
**
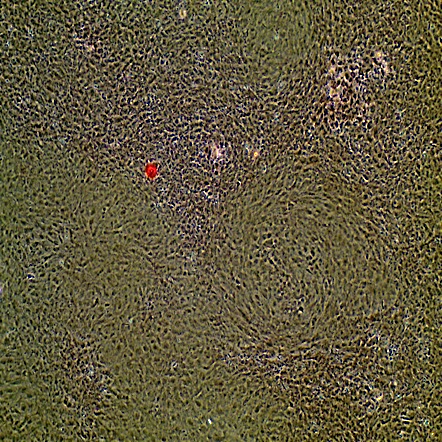

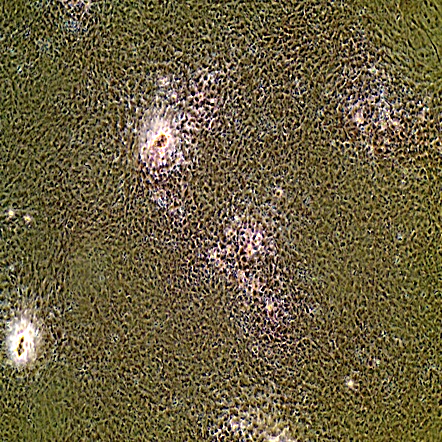


**d**

**f**

**e**

1000 µM DMOG

**Figure 2.** The effect of CoCl_2_ and DMOG on bone nodule formation. Alizarin Red staining images showed that 50 µM CoCl_2_ and 1000 µM DMOG treatments inhibited nodule formation in all glucose conditons. **a** On day 21, Alizarin Red staining (ARS) showed 50 µM CoCl_2_ not only had an inhibitory effect on nodule formation in normoxia and normal glucose (5.5 mM) but also did not restore nodule formation in **b** moderate glucose (25mM) and **c** high glucose (50 mM). **d-f** nodule formation was also completely inhibited in all 1000 µM DMOG conditions despite of the glucose levels. (Scale bar for all images is 200 μm)

**
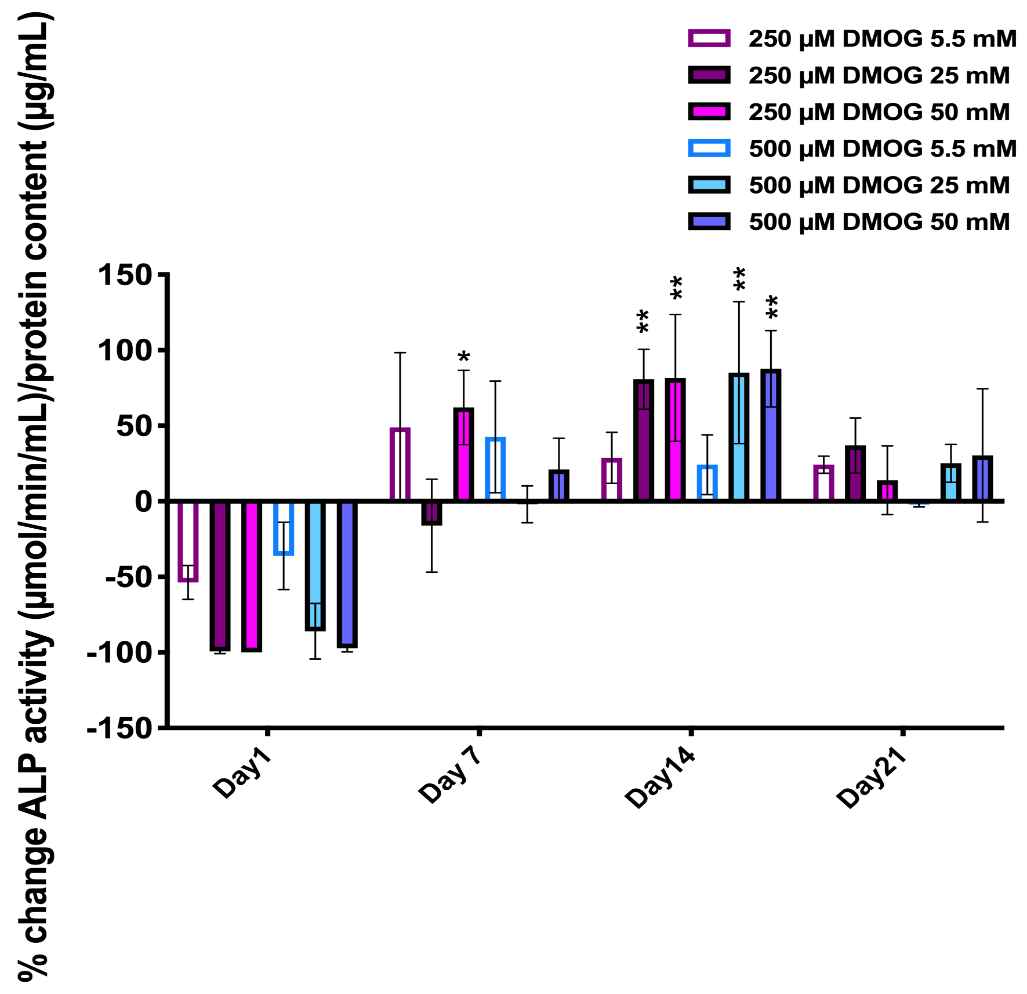

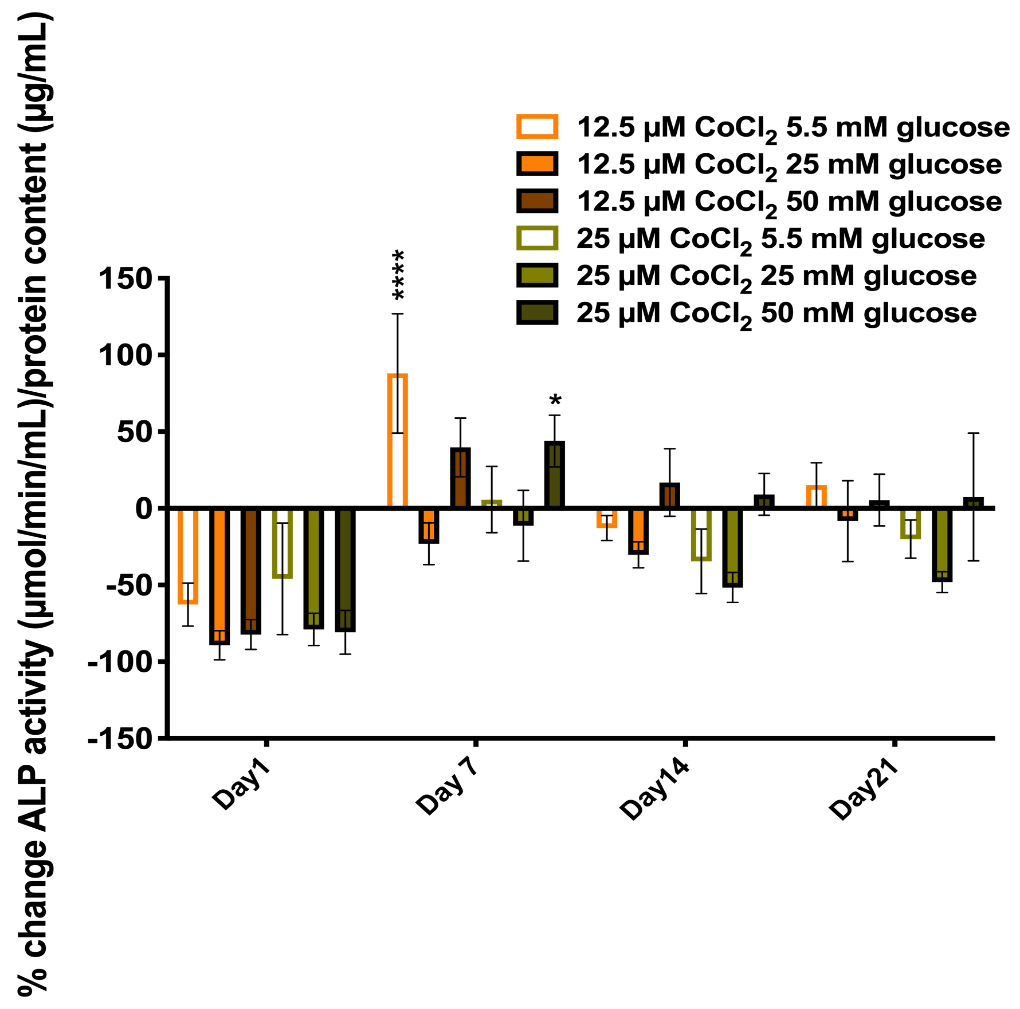
**

**b**

**a**

**Figure 3.** Neither hyperglycaemia nor hypoxia mimetics affect ALP activity. **a** Percentage change of ALP activity per unit protein compared to untreated control showed that on day 7, 12.5 μM CoCl_2_ normal glucose (5.5 mM) and 25 μM CoCl_2_ high glucose (50 mM) had higher activity than their untreated control. **b** Reduced ALP activity per unit protein was observed in all DMOG treated osteoblast compared to untreated control on day 1 but increased ALP activity on day 14. Error bars represent the SD from the mean values. *P≤0.05; **P≤0.01; ****P≤0.0001

**b**

**a**


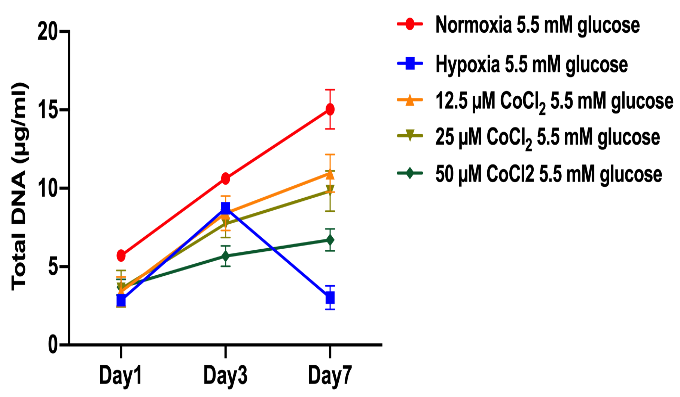

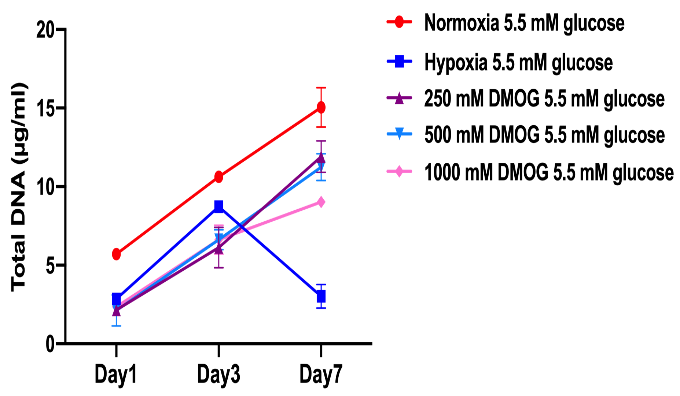


****

***

****

***

****

**d**

**c**


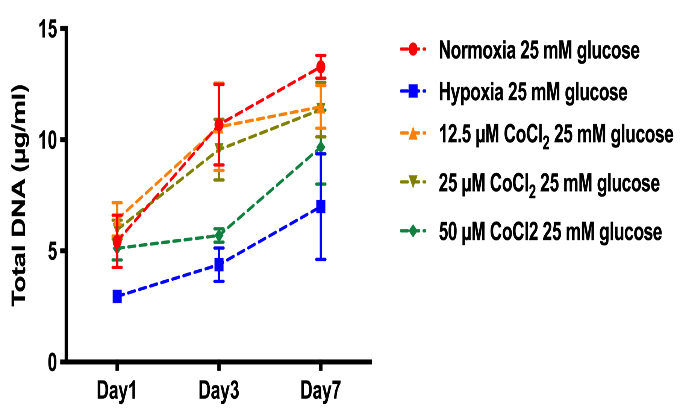

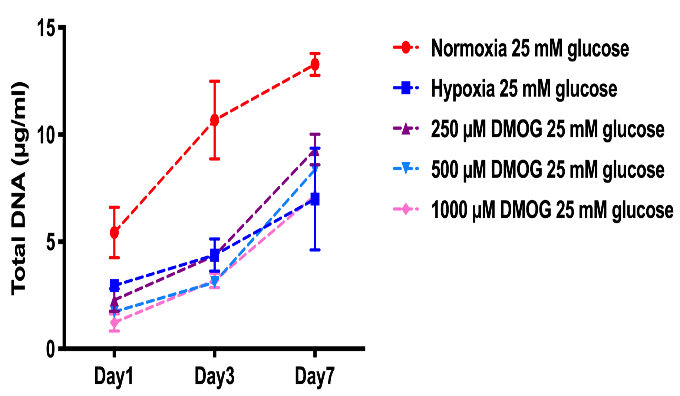


**

*

**f**

**e**


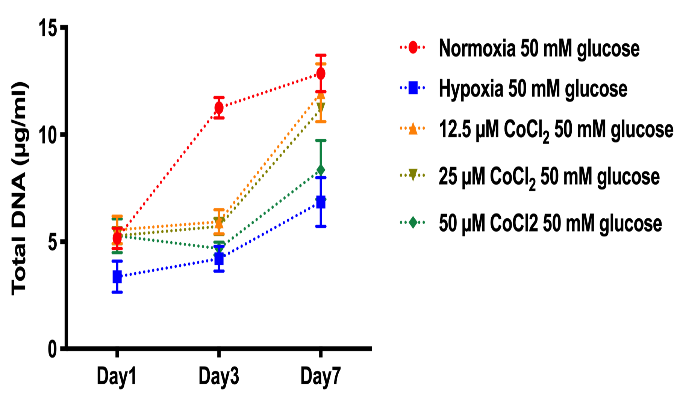

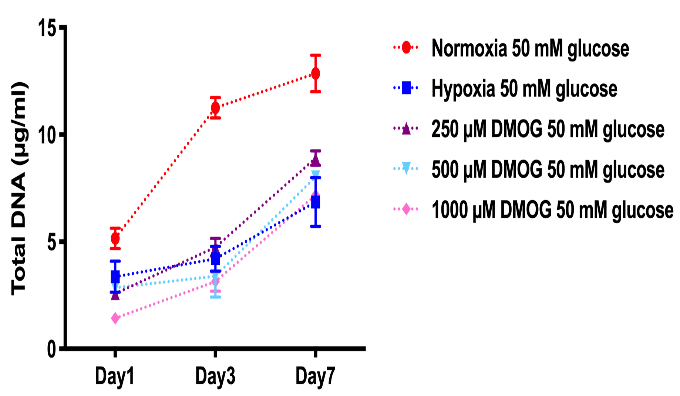


**

****

****

***

**Figure 4.** Hypoxia mimetics restored proliferation compared to prolonged hypoxia. All concentrations of **a** CoCl_2_ and **b** DMOG exhibited significantly higher proliferation than hypoxia after 7 days in normal glucose (5.5 mM). **c,d** In moderate glucose (25 mM) and **e,f** high glucose (50 mM), 12.5 and 25 µM CoCl_2_ and 250 µM DMOG increased proliferation compared to hypoxia.
